# Failed oral immunotherapy should be considered as a risk factor for fatal anaphylaxis, and omalizumab treatment considered

**DOI:** 10.1186/s13052-024-01814-7

**Published:** 2024-11-09

**Authors:** Francesca Nicolardi, Federica Corona, Laura Badina, Irene Berti, Egidio Barbi

**Affiliations:** 1 University of Trieste, Trieste, Italy; 2 Pediatric Department, Institute for Maternal and Child Health Burlo Garofolo, Trieste, Italy

**Keywords:** Fatal anaphylaxis, Oral immunotherapy, Failed immunotherapy, Omalizumab

## Abstract

Oral immunotherapy is proposed as the only active intervention to modify allergies and decrease the risk of severe reactions. However, it is crucial to note that oral immunotherapy still presents a notable failure rate and potential for severe, life-threatening outcomes. Notably, patients who discontinue oral immunotherapy may face an increased risk of fatal reactions. Omalizumab could be a viable option for patients with failed oral immunotherapy.

## Main text

Dear Editor,

We appreciate the paper by Novembre et al. [1], nicely and detailing discussing risk factors for fatal anaphylaxis, however we suggest that a relevant issue has been overlooked.

As outlined, oral immunotherapy (OIT) is currently the only active intervention that can alter the course of allergies and reduce the risk of severe anaphylaxis. However, as accurately noted by the authors, OIT still carries a significant risk of failure (approximately 20%) and can result in rare, yet severe and life-threatening reactions.

We remind that the potential for severe reactions is particularly high in those who have not successfully completed OIT and may encounter the allergen unintentionally. In this regard, we mention a study [2] in which the risk of life-threatening reactions was 3.5% in the group of patients continuing milk during OIT, compared to 6.3% in the group who had stopped treatment.

Remarkably in the latter group fatal reactions were also reported. The worrisome speculation may be that in these patients failed OIT may paradoxically act as booster. This issue is particularly relevant for adolescents, who already are at high risk, for their behaviors and outdoors life.

Moreover, we suggest that in these highly selected patients Omalizumab could be considered, as shown by a study [3] conducted on few patients, which had previously failed immunotherapy, successfully treated with omalizumab and a restart of OIT. No patients experienced severe reactions, even after omalizumab discontinuation and all increased tolerance threshold.

In conclusion, we remark that patients with failed OIT may represent a population at higher risk of fatal anaphylaxis, deserving the proposal of an Omalizumab protected OIT restart.

## Data Availability

Not applicable.

## Abbreviation

OIT: Oral immunotherapy
